# Repeatability of Teethan® indexes analysis of the masseter and anterior temporalis muscles during maximum clenching: a pilot study

**DOI:** 10.1007/s00784-023-05150-8

**Published:** 2023-07-28

**Authors:** Ovidiu Ionut Saracutu, Matteo Pollis, Edoardo Ferrari Cagidiaco, Marco Ferrari, Daniele Manfredini

**Affiliations:** Department of Biomedical Technologies, School of Dentistry, University of Siena, 53100 Siena, Italy

**Keywords:** Electromyography, Dental occlusion, Bruxism, Temporomandibular disorders, EMG

## Abstract

**Objectives:**

The aim of this study is to assess the repeatability of a surface electromyographic (EMG) device (Teethan®, Teethan S.p.A., Milan, Italy), designed for the analysis of the masseter and anterior temporalis muscles.

**Materials and methods:**

Tests were performed on a sample of 30 healthy fully dentate TMD-free individuals randomly selected. Each test consisted of two distinct recordings performed at 5-min intervals: (i) the patient is asked to clench with maximum voluntary contraction (MVC), with two cotton rolls interposed between the dental arches; (ii) the patient is asked to repeat the same clenching activity without the cotton rolls. The outcomes of the study were the EMG indices conceptualized by the manufacturing company, based on the differences between the two test conditions (i.e., clenching on cotton rolls and on dentition). Pairwise correlation analysis and ANOVA test were performed to assess the strength of correlation and the significance of differences between the results of the three trials.

**Results:**

Thirty TMD-free healthy individuals (20 females and 10 males; mean age 44 years, range 16–60 years) took part in the study. ANOVA test did not show any statistically significant difference between the three trials. The Global Index, which is the mean of the other EMG indices, showed the highest correlation values between the three trials, while some other indices showed a weak-to-medium correlation level. One out of five participants showed a coefficient of variation higher than 10%.

**Conclusions:**

The statistical analysis showed that the indices provided by the device are quite repeatable*.* However, this does not necessarily imply a specific clinical application of the device, which was here used in fully controlled experimental conditions.

**Clinical relevance:**

The clinical usefulness of the applied protocol remains questionable. Further studies should test the repeatability of EMG findings gathered with this device under various circumstances, in a more heterogeneous population.

## Introduction

Temporomandibular disorders (TMDs) represent an umbrella term that comprises a heterogenous group of musculoskeletal conditions affecting the temporomandibular joint (TMJ), the masticatory muscles, and the related structures [1]. They represent the most common cause of non-odontogenic chronic pain [2] and the second most common musculoskeletal disorder that causes pain and disability [3, 4]. TMD incidence is further exacerbated by psychological distress scenarios, such as the COVID-19 pandemic [5]. The best scientific evidence available rules out a determinant role of the occlusal features in the onset of TMDs [6–8], revealing that psychological factors represent a more important risk factor [9–11]. Moreover, some systemic comorbidity (including fibromyalgia and headache) were shown to be higher in terms of prevalence in TMD patients [12, 13]. The higher prevalence of TMD in chronic primary pain conditions related to central nervous system dysfunction, including fibromyalgia and primary headaches, can be probably explained through the phenomenon of central sensitization (mainly allodynia and hyperalgesia) [14, 15].

According to the DC/TMD, the current temporomandibular disorder standard of diagnosis is based on medical history and clinical examination [16]. However, in some patients with a doubtful diagnosis or unresponsive to treatment, instrumental examinations like magnetic resonance imaging (MRI) can help the clinician to better evaluate the TMJ status [17] and decide the best treatment plan.

In this regard, surface electromyography (EMG) is a non-invasive objective method for the analysis of masticatory muscles, via the evaluation of the myoelectric signal generated during clenching. However, the clinical usefulness of such electromyographic devices is put into question by the available scientific evidence, also because of the many technical and host-related factors that may influence the recordings at chairside. A systematic review performed by Klasser et al. demonstrates that surface EMG is not adding any additional contribution to the standard clinical evaluation [18]. A subsequent study performed by Manfredini et al. in 2011 [19] further confirms the lack of reliability of such devices, both as an adjunctive tool and as a stand-alone evaluation.

Notwithstanding, some clinicians still consider the use of surface electromyography analysis (EMG) of temporalis and masseter muscles a fundamental tool to better diagnose and discriminate muscular temporomandibular disorders [20]. Some findings showed that in TMD patients, the asymmetric contraction between the right and left muscles pairs is higher than in healthy controls. Therefore, such EMG evaluation was speculated to be useful as an additional aid in the diagnostic process, also thanks to the accessibility of the masseter and anterior temporalis muscles [21–23].

Asymmetry in the recruitment of masticatory muscles may result from a loss of function related to a condition of chronic muscles inflammation. This condition is not allowing patients to recruit all the muscle fibers and limits the muscle repair process, as well as the protein turnover mechanism [24]. Thus, asymmetric muscle contraction is potentially a consequence and not a direct cause of TMDs [25]. Nonetheless, some researchers propose the use of EMG as a screening tool for chewing muscles disorders [26].

Within this framework, a novel EMG recording device (Teethan®, Teethan S.p.A, Milan, Italy) has been conceptualized to evaluate the contraction of two pairs of masticatory muscles, the temporalis, and the masseter. Manufacturers’ claims about its potential need to assess muscle function in everyday dental practice procedures (e.g., prosthodontics, orthodontics, and orofacial pain) need to be supported by on field testing. Before stepping into the investigation of the potential clinical relevance of the device, the repeatability of findings should be assessed. Repeatability is intended as the quality of a test whereby repetition of the same protocol and procedures yields the same or closely similar results or responses each time [27].

The main aim of the study is to verify that the electromyographic registrations of the masseter and anterior temporalis muscles are repeatable within the same population, under the same circumstances. Therefore, the manuscript describes the application of the protocol proposed by the manufacturer on a sample of healthy individuals. The paper also provides suggestions for future different study designs.

## Materials and methods

### Participants

This descriptive cross-sectional study was performed on a group of individuals recruited among dental patients at the University of Siena, Siena, Italy. Participants were enrolled in 3 separated days. People in need of receiving any dental treatment were excluded from the study, as well as patients who underwent or were undergoing orthodontic treatment. The TMD Pain Screener was used to screen for the presence of pain or TMD symptoms [28]. No arbitrary age limit was set.

After filling up the informed consent and determining the eligibility according to the inclusion and exclusion criteria, researchers performed the chair-side electromyographic registrations under the supervision of a company engineer. The study was approved by the Internal Review Board of the Orofacial Pain Unit, School of Dentistry, University of Siena (#0023-2022). All individuals gave their informed consent, in accordance with the Declaration of Helsinki, and understood that they were free to withdraw from the study at any time.

### Instrumentation

The device (Teethan®, Teethan S.p.A, Milan, Italy) is composed of four EMG probes with differential electrodes that communicate through a USB receiver to convey data to a personal computer. Each probe is specific for a masticatory muscle: right and left anterior temporalis and masseter muscles. Muscle activity is assessed in microvolts. A dedicated software analyzes the received signal and elaborates the data to create indices conceptualized by the company, and described in some early studies [29], based on the differences between the two test conditions (i.e., clenching on cotton rolls and on dentition [see below]):

- POC: it is an index used to assess the symmetry of contraction standardized within the same muscle pair. It indicates the imbalance (right/left) within the examined muscle pair: in particular, the POC calculates the predominance of the right or left temporalis in the front quadrants, and that of the right or left masseter muscle, in the rear quadrants (i.e., right or left masseter—POC MM; right or left temporalis—POC TA) [29].
- BAR: it assesses the position of the occlusal barycenter. It is obtained by calculating the percentage of overlapping coefficient between the activities of the two temporals and the activities of the two masseters (unlike the POC index that compares individual analogous muscles) [30].
- TORS: this assesses the torsion attitude of the mandible in the horizontal plane when it is in occlusion with the upper jaw. It is the result of the comparison of the force couple of crossed muscle pairs: comparison between the right temporal and left masseter pair and between the left temporal and the right masseter pair [30].
- IMPACT: it indicates the muscular activity of masticatory muscles and is proportional to the bite force. The normality values of the index are over the range of 100–115% [31].
- ASIM: this index allows to compare the activity of the right muscles with that of the left ones. A positive value indicates a greater activation of the right-hand side, while a negative value indicates a greater activation of the left-hand side [32].
- Global Index: mean value of the first four indices.

### Experimental protocol

The authors adopted the protocol proposed by the manufacturer of the device. All recording trials were performed by the same operator, under the supervision of an engineer representative of the manufacturer company. For the positioning, the first two probes were located along the anterior margin of the temporalis muscle, keeping a distance of 2 cm from the zygomatic process. The other two probes were positioned in a direction that is parallel to the masseter muscle fibers course, in the central portion of the muscle, along the line joining the outside edge of the eye with the angle of the jaw.

The protocol adopted in this investigation is mediated by the original study by Ferrario et al. [30]. Each trial consists of two separated 5-s clenching tests, in which the subject is asked to perform two maximum voluntary contractions (MVC): (i) the first clenching registration is performed to obtain a reference of the EMG signal; the participant is asked to clench with two cotton rolls inserted between the arches at the level of the second premolar and the first molar. The examiners have to make sure that the patient is actively clenching the teeth. During this step, the registration is activated, and it automatically stops after 5 s. (ii) The second part of the test consists in asking the patient to perform the same clenching activity, without the cotton rolls in between the arches. A second registration of 5 s is obtained.

The probes containing the electrodes were not detached between the first and the second registration, in order to keep them in the same exact position. At the end of both registrations, a final report with all the indices described above is automatically generated by the software.

The underlying idea was that a tentatively ideal setting should be created to reduce the influence of external factors on the outcome of the measurements. Thus, we asked participants to repeat the test by keeping the same position of the body. Participants were instructed to stay seated on a chair, in a relaxed position, with the trunk perpendicular to the floor, without crossing the legs, resting the hands on the knee, and with the head straight, looking forward towards a fixed point. They were asked to not make any other type of body movement during the registration, besides clenching. Three trials of recordings (i.e., three sets of two recordings) were performed at 5-min intervals, changing the electrodes between each trial. The quality of the signal was checked according to the manufacturer’s instructions.

### Statistical analysis

The outcome variables for this cross-sectional study were the above described EMG indices, with the Global Index being considered as the primary outcome. For statistical purposes, descriptive reports of the indices were performed. ANOVA test was used to investigate for the presence of a statistically significant difference between the mean values in the EMG indices obtained in the three trials. Pairwise correlation analysis between findings of the recording trials was performed to assess the strength of correlation, which can be interpreted as a marker of repeatability of the test. The number of participants with a coefficient of variation between the three trials higher than 10% was also calculated. Significance was set at *p* < .05. The statistical analysis was performed with SPSS 26.0 (IBM, Milan, Italy) software.

## Results

The sample of this study was composed of 30 healthy fully dentate TMD free individuals (20 females and 10 males; mean age 44 years, range 16–60 years), randomly selected. Table 1 reports the age, the mean (*μ*), standard deviation (*σ*), and coefficient of variation (CV) for each electromyographic index from the 90 registrations.

**Table 1:** Age, Global Index, POC TA index, POC MM index, BAR index, TORS index, IMP ACT index ASIM index for each participant

Index	*μ*	*σ*	CV
Age	42.97	14.95	287.33
Global Index^1^ %	81.07	11.53	703.16
POC TA^2^ %	79.38	13.98	567.80
POC MM^3^ %	86.78	8.83	982.32
BAR^4^ %	78.66	17.21	457.07
TORS^5^ %	86.28	11.95	721.78
IMP ACT^6^ %	111.53	43.76	254.87
ASIM^7^ %	2.54	19.45	13.06

*μ*, mean; *σ*, standard deviation; *CV*, coefficient of variation

^1^Global Index: mean value of the first four indices

^2^POC TA: symmetry of contraction standardized within the same muscle pair, right and left temporalis

^3^POC MM: symmetry of contraction standardized within the same muscle pair, right and left masseter

^4^BAR: assessment of the position of the occlusal barycenter

^5^TORS: assessment of the torsion attitude of the mandible in the horizontal plane

^6^IMPACT: assessment of muscle activity related to the bite force

^7^ASIM: compare the activity of the right muscles with that of the left ones

ANOVA test did not find any statistically significant differences between findings of the three sets of registrations for any indices (*P* > .05) (Table 2). Pairwise correlation tests showed a wide-range of correlation values (Table 3).

**Table 2:** Results of ANOVA test

Index		Sum of squares	df	Mean square	*F*	Sig.
Global Index^1^	Between groups	45.355	2	22.677	0.148	0.862
	Within groups	13,759.806	90	152.887		
	Total	13,805.161	92			
POC TA^2^ %	Between groups	92.763	2	46.382	0.228	0.796
	Within groups	18,276.159	90	203.068		
	Total	18,368.922	92			
POC MM^3^ %	Between groups	187.347	2	93.674	1.173	0.314
	Within groups	7.189	90	79.882		
	Total	7.377	92			
BAR^4^ %	Between groups	88.006	2	44.003	0.127	0.881
	Within groups	31,283.433	90	347.594		
	Total	31,371.439	92			
TORS^5^ %	Between groups	108.268	2	54.134	0.350	0.706
	Within groups	13,928.778	90	154.764		
	Total	14,037.045	92			
IMPACT^6^ %	Between groups	3,877.256	2	1,938.628	1.006	0.370
	Within groups	173,509.132	90	1,927.879		
	Total	177,386.387	92			
ASIM^7^ %	Between groups	14.731	2	7.365	0.018	0.982
	Within groups	36,150.055	90	401.556		
	Total	36,154.786	92			

^1^Global Index: mean value of the first four indices

^2^POC TA: symmetry of contraction standardized within the same muscle pair, right and left temporalis

^3^POC MM: symmetry of contraction standardized within the same muscle pair, right and left masseter

^4^BAR: assessment of the position of the occlusal barycenter

^5^TORS: assessment of the torsion attitude of the mandible in the horizontal plane

^6^IMPACT: assessment of muscle activity related to the bite force

^7^ASIM: compare the activity of the right muscles with that of the left ones

**Table 3:** Results of the correlation tests between the different tests

Indices	Pairwise correlations
Global Index^1^ 1 and Global Index 2	0.725248
Global Index 1 and Global Index 3	0.856137
Global Index 2 and Global Index 3	0.640567
POC TA^2^ 1 and POC TA 2	0.416244
POC TA 1 and POC TA 3	0.848918
POC TA 2 and POC TA 3	0.557442
POC MM^3^ 1 and POC MM 2	0.542654
POC MM 1 and POC MM 3	0.450117
POC MM 2 and POC MM 3	0.421292
BAR^4^ 1 and BAR 2	0.800474
BAR 1 and BAR 3	0.831976
BAR 2 and BAR 3	0.731777
TORS^5^ 1 and TORS 2	0.615794
TORS 1 and TORS 3	0.882699
TORS 2 and TORS 3	0.564888
IMPACT^6^ 1 and IMPACT 2	0.532745
IMPACT 1 and IMPACT 3	0.552300
IMPACT 2 and IMPACT 3	0.295780
ASIM^7^ 1 and ASIM 2	0.531099
ASIM 1 and ASIM 3	0.688820
ASIM 2 and ASIM 3	0.426062

^1^Global Index: mean value of the first four indices

^2^POC TA: symmetry of contraction standardized within the same muscle pair, right and left temporalis

^3^POC MM: symmetry of contraction standardized within the same muscle pair, right and left masseter

^4^BAR: assessment of the position of the occlusal barycenter

^5^TORS: assessment of the torsion attitude of the mandible in the horizontal plane

^6^IMPACT: assessment of muscle activity related to the bite force

^7^ASIM: compare the activity of the right muscles with that of the left ones

The Global Index had one of the highest levels of between-trial correlation among all the indices (0.64–0.86). BAR and TORS showed a medium-to-high degree of correlation: (0.73–0.83) and (0.56–0.88). The less repeatable indices were the POC MM (0.42–0.54) and the IMPACT (0.30–0.55), which assess the symmetry of masseter muscle contraction and the overall bite force, respectively. Importantly, in 6 out of 30 participants, the coefficient of variation for the Global Index was > 10%.

## Discussion

The scope of this study is to test the repeatability of a device conceptualized for the quantitative analysis of the anterior temporalis muscles and masseter muscles during maximum voluntary clenching, which is claimed as useful to assess parameters of muscle function that might have an impact on everyday dental procedures. Such an evaluation is indeed a step before investigating the clinical validity of the device in terms of diagnostic usefulness, viz., it is fundamental to verify that the tool is providing the same results, in the same participants, under the same circumstances. Considering that at the moment, no literature is available on the instrument; the researchers fully adopted the utilization protocol proposed by the manufacturer.

In addition to this, it is known that operator experience plays an important role in the reproducibility of EMG measurements [33], as well as the correct position of the electrodes [34]. To make sure that the operator is respecting the protocol, an engineer of the company was present during the performance of the registrations. To avoid that the different conductivity of the skin could change the amplitude of the bio-signal, by keepings the probes attached for various minutes, electrodes were changes between the three trials made by each patient.

The results of the paper show that the indices provided by the device have a wide range of variability as for the repeatability values, with some being more repeatable than others. In particular, the Global Index, which is defined by the manufacturer as the main index that summarizes together all the recorded results, shows one of the highest degrees of repeatability.

This protocol, evaluating muscle asymmetry during maximum voluntary clenching, has been proposed, amongst the others by Ferrario et al. The authors elaborated a method for the within-subject and during time standardization of the EMG potential to evaluate the muscle symmetry during MVC. The POC index, calculated by the device tested in this study, quantifying the asymmetric muscle contraction, was implemented for the first time [30]. The same protocol was then conceptualized by Tartaglia et al. as a reliable tool to discriminate among the various categories of the RDC/TMD [22].

Several studies have tested the clinical use of surface EMG of the masseter and anterior temporalis muscles, adopting different protocols. In 2005 Castroflorio et al. [35], performed on a group of patients with TMD and a control group without TMD, a study showing a statistically significant reproducibility of EMG signals. The authors also concluded that the proper location for the electrodes is critical for obtaining an adequate analysis of muscles contraction. Suvinen et al. [36] showed that maximum voluntary clenching (MVC) could be reproduced from day 1 to day 2 and therefore were most repeatable of the dynamic tasks compared with opening and closing movement data.

The same authors, however, found more variability of the data during the dynamic recordings performed on the same patients, showing some surface EMG limitations. The same conclusions were reached by Yeong-Gwam Im et al. [37], in a sample of healthy men in which the variability of the static measurements obtained on the same patients was very small.

This paper focuses only on the repeatability of the surface EMG recordings, which represents a first fundamental step before drawing any speculation related to its clinical validity. Dental devices such as postural platform, proposed by some manufacturers and practitioners as a valid diagnostic tool in clinical practice, has been proven by many scientific studies to be not reliable nor repeatable [38, 39]. Several studies tried to test the use of EMG as a diagnostic tool to discriminate between TMD patients and healthy individuals, and they reached contradictory results.

In a 2012 paper by De Felicio CM et al. [21], surface EMG was used to find possible differences in masticatory muscle activity during clenching between 42 TMD patients and a statistically enough number of healthy controls, 18. TMD patients showed more asymmetric muscle contraction between right and left pairs than controls. These results are in agreement with the study of Tartaglia et al. [23]. TMD patients had a more asymmetric activity of the anterior temporalis muscle.

In contrast to these findings, the study performed by Manfredini et al. [19] on surface EMG was not able to discriminate between TMD patients and healthy controls. The only parameter that was different between the two groups was the one related to the masseter and anterior temporalis muscles activity during clenching, significantly lower in the TMD group. Instead, the authors did not find any statistically significant difference in the asymmetrical contraction of the muscles between the two groups. The same group of researchers performed a similar study design in a sample of 30 TMD patients having muscle pain only on one side of the face [25]. The paper is aimed at investigating if surface EMG is able to detect any difference in muscle activity, either at rest or during clenching. The authors concluded that no difference was present in EMG activity between painful and non-painful sites. A recent systematic review performed by Barros et al. tried to shed light on the topic. Authors come to the conclusion that, due to the different methodological approach related to analysis and processing of the data, no evidence supports the capability of sEMG to discriminate TMD patients from healthy control. The results of these studies put into question the clinical usefulness of surface electromyography [40].

Temporomandibular disorders comprehend masticatory muscle dysfunctions, which can alter the correct function of the masticatory system [16]. It is well known in the literature that psychological factors represent the main risk factor for the onset of TMD (Axis II from DC/TMD) [41, 42]. Therefore, the eventual functional limitation can be seen more as a consequence of prolonged clenching activity and psychological risk factors, than as a cause of asymmetric recruitment of masticatory muscle fibers [43, 44]. Considering the discordance of the studies found in the literature, the use of surface EMG as a screening tool for TMD patients’ needs to be better refined and reconceptualized, with focus on the same repeatability and reliability studies that have been performed in the field of body posture assessment.

Muscle fatigue is a phenomenon that can impair muscle contraction and decrease in maximal force. However, considering that participants remains in MVCs for 5 s, repeated for six times in a time span of 15 min, it is unluckily that fatigue significantly impact registrations in TMD-free participants.

This paper’s findings showed that the device provides a very wide range of correlation between three trials repeated in the same individual. ANOVA test confirmed that no statistically significant difference exists between the three sets of recordings performed on the same patient. However, it must be borne in mind that the tests were performed under very specific and strict criteria, which cannot be compared to the everyday chairside use that is expected by average clinicians. Registrations were conducted on the same day, 5 min apart from each other, without detaching the probes from the participants’ skin and asking the participants to remain in a fixed and static position. It must be noted that even by adopting a strict experimental protocol, 6 participants on 30 showed to have a coefficient of variation higher than 10% in the Global Index, which resulted to be the most repeatable index according to pairwise-correlation test. This finding prevents the authors from making any inference on the clinical applicability of the device in the dental practice. Other types of study design should challenge the repeatability of the device under different circumstances, that consider the variable of time, perhaps testing the repeatability of the device on the same participants 1 day or 1 week apart. Moreover, the authors tested the surface EMG only on healthy individuals. Further studies should verify if the device is repeatable in patients affected by orofacial pain conditions and if it provides different indices compared to healthy controls.

## Conclusions

The paper presents pilot data testing the repeatability of a novel surface EMG device for the recording of the masseter and anterior temporalis muscle during clenching. The main limits of this study are represented by the sample population, the specific criteria that patients were asked to adopt during the performance of the registrations, and the lack of follow-up. The results show that the indices provided by the device are quite repeatable, with the Global Index, which summarize all the gathered data, being one of the most repeatable measures. The measurements were performed under strict and precise control of the possible confounding external factors, on a population of healthy subjects, in a condition that actually does not resemble the everyday use in a dental setting. Further studies are needed to test the correct functioning of the device, putting into question the repeatability of recordings over time (i.e., different days, electrodes repositioning) as well as the reliability in detecting differences between patients with specific conditions.

## References

[1] Okeson JP (2008). The classification of orofacial pains. Oral Maxillofac Surg Clin North Am.

[2] List T, Jensen RH (2017). Temporomandibular disorders: old ideas and new concepts. Cephalalgia.

[3] Valesan LF, Da-Cas CD, Réus JC, Denardin ACS, Garanhani RR, Bonotto D, Januzzi E, de Souza BDM (2021). Prevalence of temporomandibular joint disorders: a systematic review and meta-analysis. Clin Oral Investig.

[4] Jin LJ, Lamster IB, Greenspan JS, Pitts NB, Scully C, Warnakulasuriya S (2016). Global burden of oral diseases: emerging concepts, management and interplay with systemic health. Oral Dis.

[5] Colonna A, Guarda-Nardini L, Ferrari M, Manfredini D (2021). COVID-19 pandemic and the psyche, bruxism, temporomandibular disorders triangle. Cranio.

[6] Manfredini D, Lombardo L, Siciliani G (2017). Temporomandibular disorders and dental occlusion. A systematic review of association studies: end of an era?. J Oral Rehabil.

[7] Manfredini D, Castroflorio T, Perinetti G, Guarda-Nardini L (2012). Dental occlusion, body posture and temporomandibular disorders: where we are now and where we are heading for. J Oral Rehabil.

[8] Manfredini D, Perinetti G, Stellini E, Di Leonardo B, Guarda-Nardini L (2015). Prevalence of static and dynamic dental malocclusion features in subgroups of temporomandibular disorder patients: implications for the epidemiology of the TMD-occlusion association. Quintessence Int.

[9] Guarda-Nardini L, Pavan C, Arveda N, Ferronato G, Manfredini D (2012). Psychometric features of temporomandibular disorders patients in relation to pain diffusion, location, intensity and duration. J Oral Rehabil.

[10] Manfredini D, Bandettini di Poggio A, Cantini E, Dell'Osso L, Bosco M (2004). Mood and anxiety psychopathology and temporomandibular disorder: a spectrum approach. J Oral Rehabil.

[11] Manfredini D, Cerea S, Pavan C, Guarda-Nardini L (2018). Personality traits are potentially associated with the presence of chronic temporomandibular joint pain in patients without effusion as determined by T-2 weighted magnetic resonance. Cranio.

[12] Ferrillo M, Migliario M, Marotta N, Fortunato F, Bindi M, Pezzotti F, Ammendolia A, Giudice A, Foglio Bonda PL, de Sire A (2023). Temporomandibular disorders and neck pain in primary headache patients: a retrospective machine learning study. Acta Odontol Scand.

[13] Moreno-Fernández AM, Jiménez-Castellanos E, Iglesias-Linares A, Bueso-Madrid D, Fernández-Rodríguez A, de Miguel M (2017). Fibromyalgia syndrome and temporomandibular disorders with muscular pain. A review. Mod Rheumatol.

[14] Ferrillo M, Giudice A, Marotta N, Fortunato F, Di Venere D, Ammendolia A, Fiore P, de Sire A (2022). Pain management and rehabilitation for central sensitization in temporomandibular disorders: a comprehensive review. Int J Mol Sci.

[15] Vale Braido GVD, Svensson P, Dos Santos PJ, Mercante FG, Fernandes G, de Godoi Gonçalves DA (2023). Are central sensitization symptoms and psychosocial alterations interfering in the association between painful TMD, migraine, and headache attributed to TMD?. Clin Oral Investig.

[16] Schiffman E, Ohrbach R, Truelove E, Look J, Anderson G, Goulet JP, List T, Svensson P, Gonzalez Y, Lobbezoo F, Michelotti A, Brooks SL, Ceusters W, Drangsholt M, Ettlin D, Gaul C, Goldberg LJ, Haythornthwaite JA, Hollender L, Jensen R, John MT, De Laat A, de Leeuw R, Maixner W, van der Meulen M, Murray GM, Nixdorf DR, Palla S, Petersson A, Pionchon P, Smith B, Visscher CM, Zakrzewska J, Dworkin SF, International RDC/TMD Consortium Network, International Association for Dental Research; Orofacial Pain Special Interest Group, International Association for the Study of Pain (2014). Diagnostic criteria for temporomandibular disorders (DC/TMD) for clinical and research applications: recommendations of the International RDC/TMD Consortium Network* and Orofacial Pain Special Interest Group†. J Oral Facial Pain Headache.

[17] Gauer RL, Semidey MJ (2015). Diagnosis and treatment of temporomandibular disorders. Am Fam Physician.

[18] Klasser GD, Okeson JP (2006). The clinical usefulness of surface electromyography in the diagnosis and treatment of temporomandibular disorders. J Am Dent Assoc.

[19] Manfredini D, Cocilovo F, Favero L, Ferronato G, Tonello S, Guarda-Nardini L (2011). Surface electromyography of jaw muscles and kinesiographic recordings: diagnostic accuracy for myofascial pain. J Oral Rehabil.

[20] Cooper BC (2004). Parameters of an optimal physiological state of the masticatory system: the results of a survey of practitioners using computerized measurement devices. Cranio.

[21] De Felício CM, Ferreira CL, Medeiros AP (2012). Rodrigues Da Silva MA, Tartaglia GM, Sforza C. Electromyographic indices, orofacial myofunctional status and temporomandibular disorders severity: a correlation study. J Electromyogr Kinesiol.

[22] Tartaglia GM (2008). Moreira Rodrigues da Silva MA, Bottini S, Sforza C, Ferrario VF. Masticatory muscle activity during maximum voluntary clench in different research diagnostic criteria for temporomandibular disorders (RDC/TMD) groups. Man Ther.

[23] Tartaglia GM, Lodetti G, Paiva G, De Felicio CM, Sforza C (2011). Surface electromyographic assessment of patients with long lasting temporomandibular joint disorder pain. J Electromyogr Kinesiol.

[24] Howard EE, Pasiakos SM, Blesso CN, Fussell MA, Rodriguez NR (2020). Divergent roles of inflammation in skeletal muscle recovery from injury. Front Physiol.

[25] Manfredini D, Cocilovo F, Stellini E, Favero L, Guarda-Nardini L (2013). Surface electromyography findings in unilateral myofascial pain patients: comparison of painful vs. non painful sides. Pain Med.

[26] Soikher M, Soikher M, Slavicek G (2010). Clinical application of electromyography in patients with myofascial pain syndrome: a case report. J Stomat Occ Med.

[27] A Dictionary of Public Health (2018) Oxford University Press. 10.1093/acref/9780191844386.001.0001

[28] Gonzalez YM, Schiffman E, Gordon SM, Seago B, Truelove EL, Slade G, Ohrbach R (2011). Development of a brief and effective temporomandibular disorder pain screening questionnaire: reliability and validity. J Am Dent Assocs.

[29] Ferrario VF, Sforza C, Colombo A, Ciusa V (2000). An electromyographic investigation of masticatory muscles symmetry in normo-occlusion subjects. J Oral Rehabil.

[30] Ferrario VF, Tartaglia GM, Galletta A, Grassi GP, Sforza C (2006). The influence of occlusion on jaw and neck muscle activity: a surface EMG study in healthy young adults. J Oral Rehabil.

[31] Ferrario VF, Sforza C, Zanotti G, Tartaglia GM (2004). Maximal bite forces in healthy young adults as predicted by surface electromyography. J Dent.

[32] Naeije M, McCarroll RS, Weijs WA (1989). Electromyographic activity of the human masticatory muscles during submaximal clenching in the inter-cuspal position. J Oral Rehabil.

[33] Ferrario VF, Sforza C, D’Addona A, Miani A (1991). Reproducibility of electromyographic measures: a statistical analysis. J Oral Rehabil.

[34] Iwasaki S, Tokunaga T, Baba S, Tanaka M, Kawazoe T (1990). Noninvasive estimation of the location of the end plate in the human masseter muscle using surface electromyograms with an electrode array. J Osaka Dent Univ.

[35] Castroflorio T, Icardi K, Torsello F, Deregibus A, Debernardi C, Bracco P (2005). Reproducibility of surface EMG in the human masseter and anterior temporalis muscle areas. Cranio.

[36] Suvinen TI, Malmberg J, Forster C, Kemppainen P (2009). Postural and dynamic masseter and anterior temporalis muscle EMG repeatability in serial assessments. J Oral Rehabil.

[37] Im YG, Han SH, Park JI, Lim HS, Kim BG, Kim JH (2017). Repeatability of measurements of surface electromyographic variables during maximum voluntary contraction of temporalis and masseter muscles in normal adults. J Oral Sci.

[38] Perinetti G (2007). Temporomandibular disorders do not correlate with detectable alterations in body posture. J Contemp Dent Pract.

[39] Perinetti G, Marsi L, Castaldo A, Contardo L (2012). Is postural platform suited to study correlations between the masticatory system and body posture? A study of repeatability and a meta-analysis of reported variations. Prog Orthod.

[40] Massaroto Barros B, Biasotto-Gonzalez DA, Bussadori SK, Gomes CAFP, Politti F (2020). Is there a difference in the electromyographic activity of the masticatory muscles between individuals with temporomandibular disorder and healthy controls? A systematic review with meta-analysis. J Oral Rehabil.

[41] Manfredini D, Winocur E, Ahlberg J, Guarda-Nardini L, Lobbezoo F (2010). Psychosocial impairment in temporomandibular disorders patients. RDC/TMD axis II findings from a multicentre study. J Dent.

[42] Canales GT, Guarda-Nardini L, Rizzatti-Barbosa CM, Conti PCR, Manfredini D (2019). Distribution of depression, somatization and pain-related impairment in patients with chronic temporomandibular disorders. J Appl Oral Sci.

[43] Manfredini D, Lobbezoo F (2021). Sleep bruxism and temporomandibular disorders: A scoping review of the literature. J Dent.

[44] Câmara-Souza MB, Bracci A, Colonna A, Ferrari M, Rodrigues Garcia RCM, Manfredini D (2023). Ecological momentary assessment of awake bruxism frequency in patients with different temporomandibular disorders. J Clin Med.

